# Supplementary material for: Chronic miR‐29 antagonism promotes favorable plaque remodeling in atherosclerotic mice
Source: EMBO Mol Med. 2016 May 2;8(6):643–53. doi: 10.15252/emmm.201506031 (PMC4888854; doi:10.15252/emmm.201506031)
Supplement: Supplementary file 1 — Appendix [file EMMM-8-643-s001.pdf]

Table of Contents

APPENDIX TABLE S1A-B ---------- **I**

APPENDIX TABLE S1A-B-LEGEND ---------- **II**

A

Protein	Bayes Factor	Fold Change	FDR
COL5A1	123.958	-1.051	0
FKB10	36.068	-0.778	0
TPM4	16.657	-0.732	0
FLNC	13.43	0.721	0
C1RA	488.009	0.528	0
CATB	15.621	0.431	0
COL2	72.411	0.699	0
COL3A1	22274.654	-0.622	0
COL5A2	236.543	-0.492	0
FLNB	314.511	-0.69	0
K1C10	13.819	0.626	0
PTPRK	60.975	0.686	0
SPRC	645.271	-0.674	0
TPM1	9.544	-0.411	0
FBN1	6.715	-0.428	0.00002
FUBP1	3.632	-0.421	0.00834
MINP1	3.774	0.553	0.01437
CANT1	3.667	0.415	0.01784
FAM3C	3.363	0.61	0.03328
BTD	3.253	0.566	0.04172
CADH2	8.843	0.396	0
COL1A1	7412.09	-0.366	0
CS1A	6512.823	0.353	0
EF1A1	14.033	-0.349	0
QSOX1	26.64	0.328	0
SPA3N	12.164	0.388	0
KPYM	6.723	0.3	0.00007
IC1	6.455	0.293	0.00011
ECM1	6.325	0.363	0.00013
SVEP1	6.293	0.248	0.00014
EGFR	5.264	0.397	0.00083
FBLN4	5.177	0.369	0.00097
TIMP2	4.765	0.306	0.00207
NID1	4.27	-0.292	0.00211
CO6A2	4.19	-0.18	0.0025
FLNA	3.725	-0.227	0.00679
K2C5	4.127	0.275	0.00709
PRDX2	4.116	0.308	0.00726
HSP7C	3.476	-0.217	0.01179
PGCP	3.663	0.399	0.01802
LTBP4	3.273	0.399	0.03997

B

Protein	Bayes Factor	Fold Change	FDR
COL5A2	3343665125	3.713	0
COL4A2	1019.147	2.714	0
NOV	7218.257	2.838	0
IGF2	1103.792	2.708	0
MMP2	77977.623	2.489	0
MOES	1033.92	-2.777	0
FLNA	120578907.5	-2.716	0
TKT	2535.467	-1.722	0
FLNB	3192.723	-1.992	0
CADH2	672847.005	-2.208	0
COL1A2	2.76621E+12	0.982	0
COL1A1	1.66559E+15	1.463	0
COL3A1	2400316249	1.879	0
KPYM	7979866942	-1.568	0
CFAH	2450.679	0.589	0
HSP7C	9300912.091	-1.181	0
COL6A2	577.144	0.563	0
VINC	2522695.075	-1.557	0
PCOC1	548.756	0.547	0
G3P	907.364	-0.93	0
EF1A1	81564.749	-1.028	0
LG3BP	4772.107	-0.607	0
SPA3N	306.065	0.534	0
ALDOA	1049.922	-1.173	0
PROF1	4435.35	-0.902	0
LTBP4	6036.021	-1.025	0
COL2	490.619	1.072	0
RNAS4	572.929	0.786	0
PRDX1	430.841	-0.592	0.00001
VCAM1	325.379	-0.677	0.00003
SPRC	91.685	0.734	0.00009
ACTB	222.582	-0.419	0.00012
PGBM	70.808	0.592	0.00022
MMP3	67.621	2.053	0.00026
MDHM	162.029	-0.708	0.00036
GDIR1	152.506	-0.616	0.00045
AATC	131.897	-2.541	0.00072
ROA2	127.109	-0.636	0.00082
IBP7	38.786	2.384	0.00161
TPIS	98.924	-0.637	0.0018
UBE2N	81.727	-2.394	0.00321
PLTP	30.634	0.503	0.00328
VEGFD	26.839	0.712	0.0048
TCO2	26.357	0.572	0.00506
FBN1	22.831	2.365	0.00754
PPIC	21.393	0.716	0.00899
TETN	21.249	2.224	0.00916
PTPRK	44.425	-0.813	0.01704
COF1	31.881	-0.636	0.0376
FINC	1032.018	-0.27	0
QSOX1	47.446	0.327	0.00085
CO3	41.348	0.318	0.00132
CO6A1	17.253	0.208	0.01574

Appendix Table S1: Comprehensive protein list from murine VSMC secretome following miR-29 manipulation

Statistically significant proteins are listed in ascending order of FDR from control versus (A) antimiR-29 and (B) mimic-29-treated VSMCs. (FDR=False Discovery Rate)
