# Supplementary material for: Chronic miR‐29 antagonism promotes favorable plaque remodeling in atherosclerotic mice
Source: EMBO Mol Med. 2016 May 2;8(6):643–53. doi: 10.15252/emmm.201506031 (PMC4888854; doi:10.15252/emmm.201506031)
Supplement: Supplementary file 2 — Expanded View Figures PDF [file EMMM-8-643-s002.pdf]

Expanded View Figures

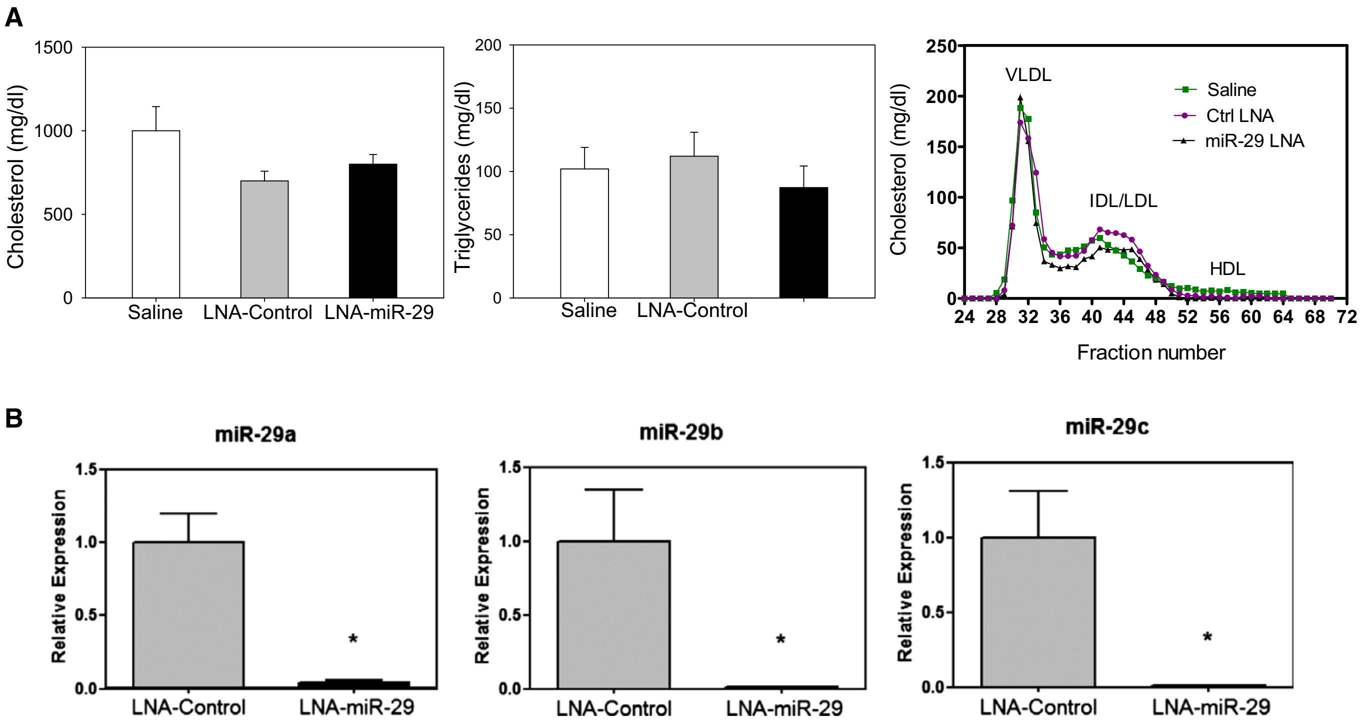

Figure EV1. LNA-miR-29 does not induce changes in lipid profile, but targets the miR-29 family.

A Total cholesterol (left panel), triglycerides (middle panel), and lipoprotein profile (right panel) from saline-, LNA-control-, or LNA-miR-29-injected ApoE$^{-/-}$ mice (n = 6, 9, 13 TC and TG, n = 9, 4, 5, profile). Data represent mean ± SEM

B Carotid arteries from LNA-control-treated (gray bar) and LNA-miR-29-treated (black bar) mice (both dosed at 4 mg/kg for 14 weeks in atherosclerosis study) were evaluated by qPCR for miR-29a (left panel), miR-29b (middle panel), and miR-29c (right panel) expression levels. Data represent mean ± SEM, *P < 0.001 versus LNA-control tissue, n = 3 measurements per treatment group repeated twice. Level of significance was determined using one-way ANOVA with Bonferroni's post-test.

 © 2016 The Authors

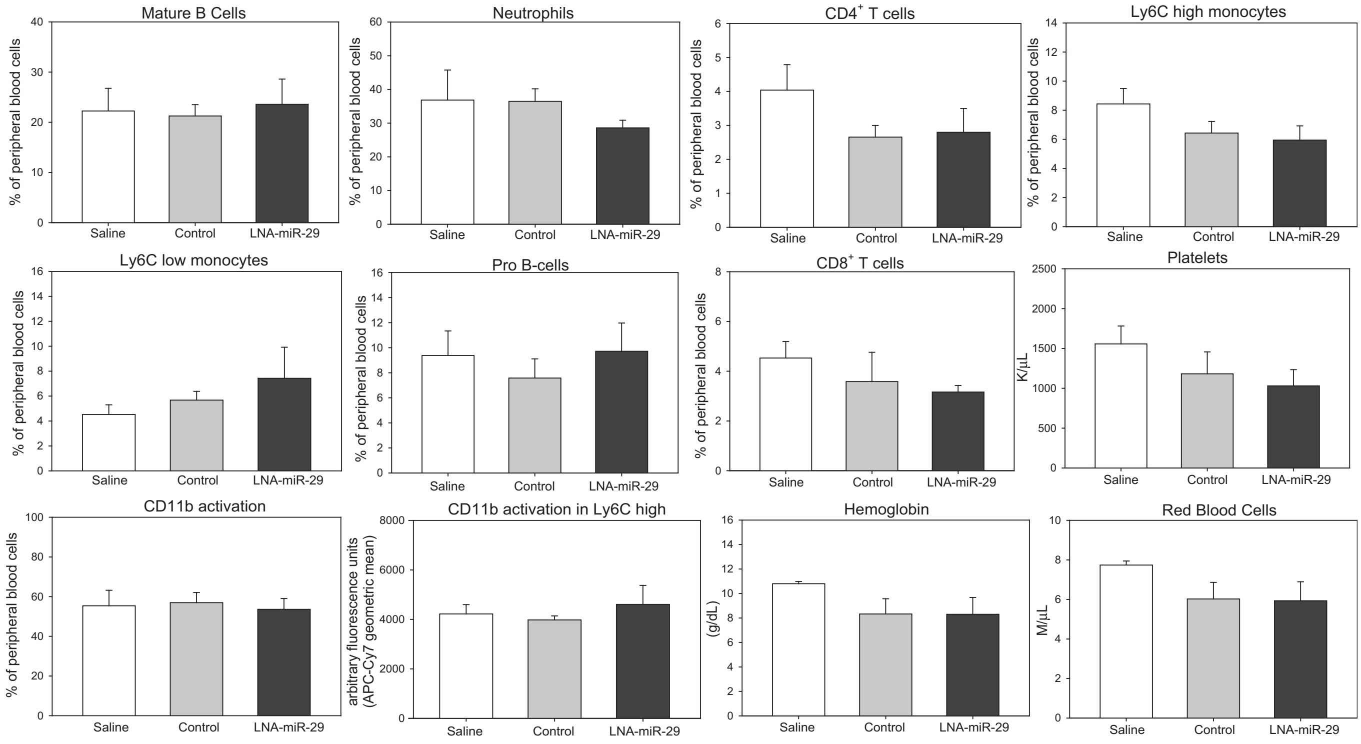

Figure EV2. LNA-miR-29 does not induce changes in circulating blood parameters.
Flow cytometry analyses of peripheral blood cell parameters in retro-orbital blood of study mice (n = 4). Data represent mean ± SEM. Level of significance was determined using one-way ANOVA with Bonferroni's post-test.

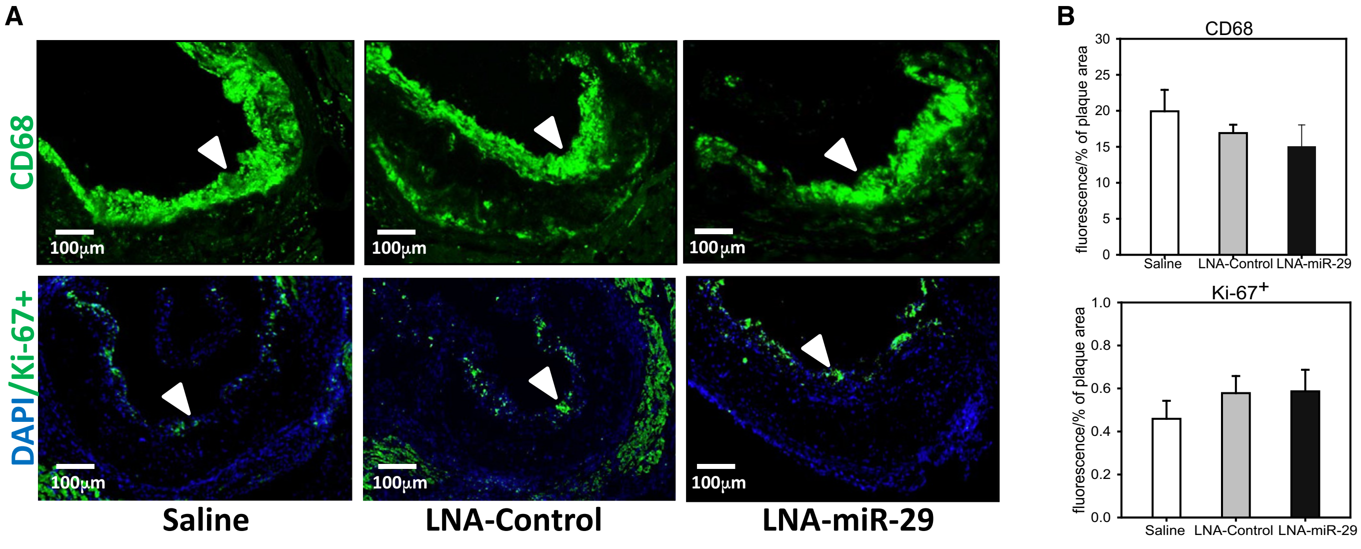

Figure EV3. LNA-miR-29 does not influence macrophage number or cell proliferation in plaques.

A Representative immunofluorescence (IF) staining of aortic root lesions from saline-, LNA-control-, and LNA-miR-29-injected ApoE$^{-/-}$ mice, shown at 10× magnification, denoted by white arrowheads. Top panel shows CD68 (green), bottom panel is Ki-67^{+} (green) costained with DAPI (blue). Scale bar, 100 μm.

B IF in root lesions quantified for CD68 (middle) and Ki-67^{+} (bottom) graphed as percent of plaque area. Data represent mean ± SEM. IF images in (A) are representative of data in (B) quantified from 3- to 6-μm serial sections confirmed from animals in independent cohorts, n = 9, 13, 15.

© 2016 The Authors

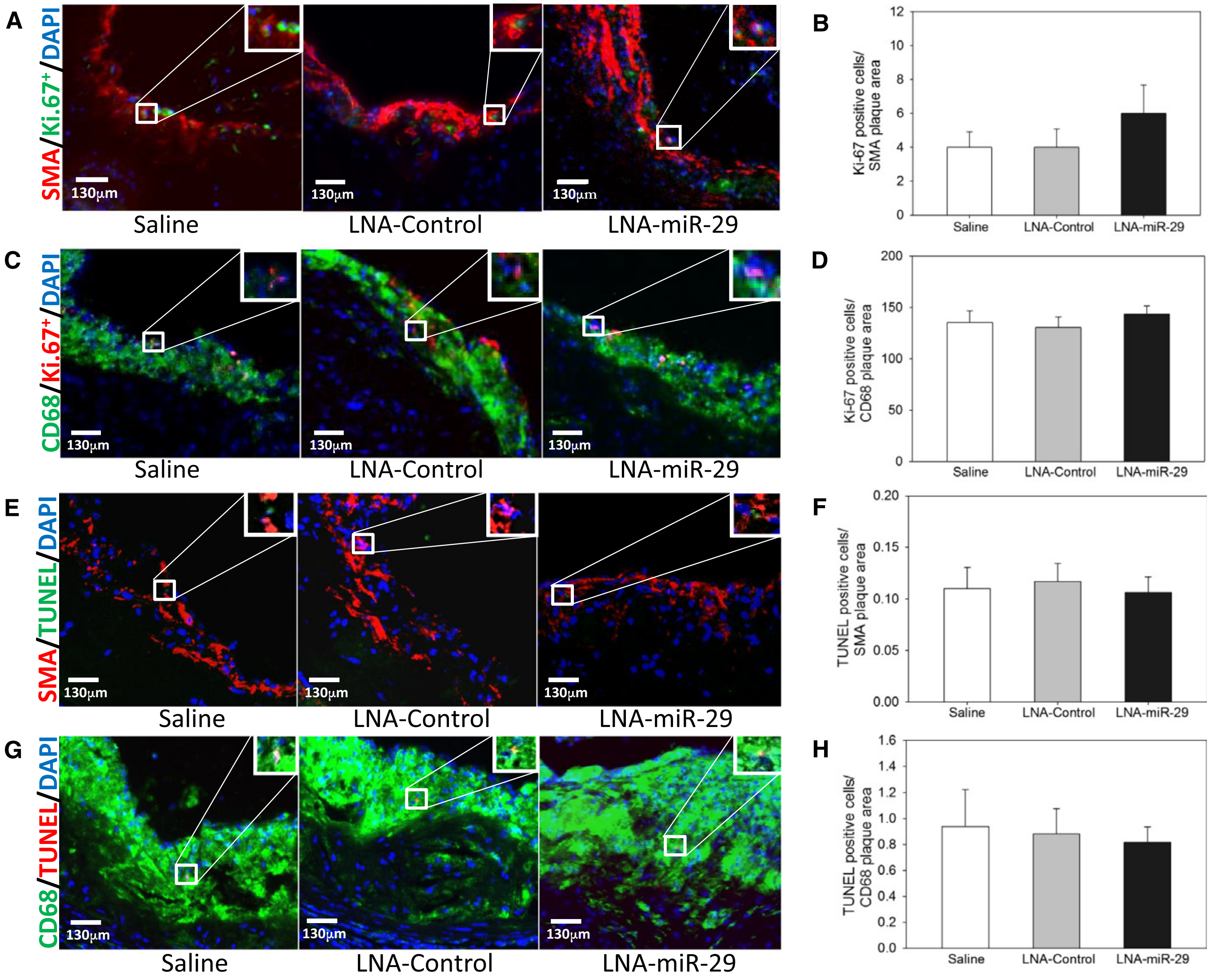

Figure EV4. LNA-miR-29 does not affect proliferation or apoptosis of VSMC or macrophages in plaques.
Immunofluorescence (IF) staining of aortic root lesions from saline-, LNA-control-, and LNA-miR-29-injected $ApoE^{-/-}$ mice, shown at 20× magnification, costaining represented in white boxes with magnified inset image of cell with both markers present (A, C, E, G).

- A Panel of images shows representative images of smooth muscle actin (red), Ki-67^{+} (green), and DAPI (blue) costaining.
- B Immunofluorescence in root lesions is quantified for cells that are Ki-67^{+} (green) and DAPI (blue)-positive within the SMA (red) plaque area.
- C Panel of images shows representative images of CD68 (green), Ki-67^{+} (red), and DAPI (blue) costaining.
- D Immunofluorescence in root lesions is quantified for cells that are Ki-67^{+} (green) and DAPI (blue)-positive within the CD68 (green) plaque area.
- E Panel of images shows representative images of smooth muscle actin (red), $TUNEL^{+}$ (green), and DAPI (blue) costaining.
- F Immunofluorescence in root lesions is quantified for cells that are $TUNEL^{+}$ (green) and DAPI (blue)-positive within the SMA (red) plaque area.
- G Panel of images shows representative images of CD68 (green), $TUNEL^{+}$ (red), and DAPI (blue) costaining.
- H Immunofluorescence in root lesions is quantified for cells that are $TUNEL^{+}$ (red) and DAPI (blue)-positive within the CD68 (green) plaque area.

Data information: IF images in (A, C, E, G) are representative of data in (B, D, F, H) quantified from 3- to 6-μm serial sections confirmed from animals in independent cohorts, $n = 9, 13, 15$. Data represent mean ± SEM. Level of significance was determined using one-way ANOVA with Bonferroni's post-test. Scale bars, 130 μm.

© 2016 The Authors

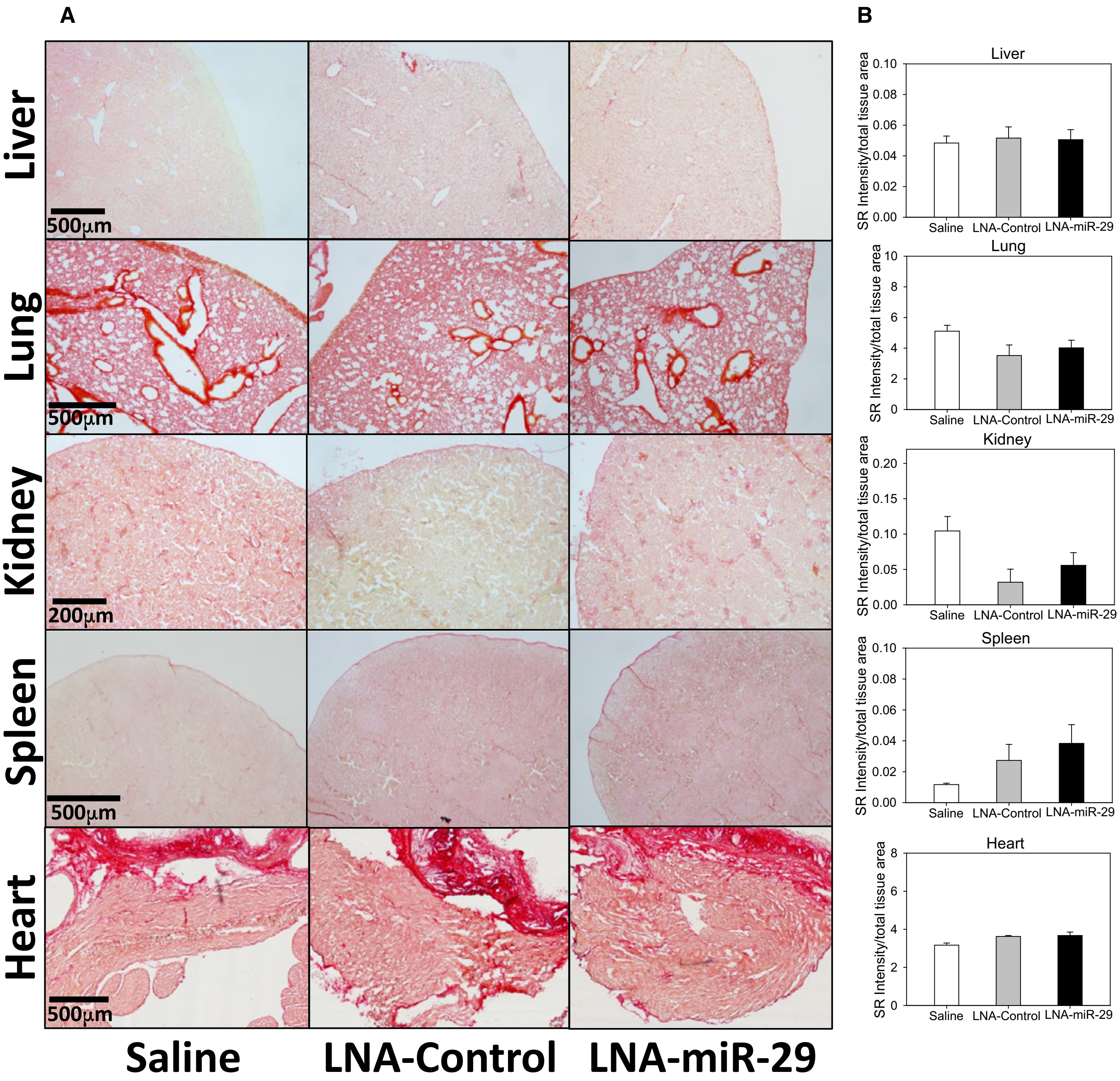

Figure EV5. LNA-miR-29 treatment does not induce organ fibrosis.

A Representative images of fibrosis analysis by picrosirius red staining in solid organs. Scale bars, 500 µm (liver, lung, spleen, and heart) and 200 µm (kidney).

B Intensity quantification of picrosirius red staining collagen in various tissues expressed as a percentage of total area. Data graphed represent mean + SEM, level of significance determined using one-way ANOVA with Bonferroni's post-test. Data information: Stained images (A) and quantification (B) are representative of 3- to 6-µm serial sections from each animal at 4× magnification, *n* = 9, 13, 15.

© 2016 The Authors
